# Examining the Roles of Psychological Inflexibility and Distress Tolerance on Cigarette Dependence and Binge Drinking among Individuals who Co-use Cannabis for Therapeutic Purposes

**DOI:** 10.1007/s11469-025-01594-z

**Published:** 2025-11-21

**Authors:** Silvana Agterberg, Mary Barna Bridgeman, Benjamin Billingsley, Rachel L. Rosen, Thomas W. O’Kane, Marc L. Steinberg

**Affiliations:** 1 Montefiore Medical Center, Bronx, NY, USA; 2 Ernest Mario School of Pharmacy, Rutgers University, Piscataway, NJ, USA; 3 The Concord Center, Concord, MA, USA; 4 Department of Psychiatry, Massachusetts General Hospital, Boston, MA, USA; 5 Rutgers University Behavioral Health Care, Piscataway, NJ, USA; 6 Rutgers Robert Wood Johnson Medical School, 317 George Street, Suite 105, New Brunswick, NJ 08901, USA

**Keywords:** Cannabis, Experiential avoidance, Medical marijuana, Nicotine, Psychological flexibility, Tobacco

## Abstract

The aims of this study were to examine associations between psychological inflexibility and (1) cigarette dependence among individuals who co-use cannabis and combustible cigarettes, and (2) binge drinking among individuals who co-use cannabis and alcohol. Adults attending a medical marijuana dispensary (N = 697) completed a survey examining cannabis use, cigarette smoking, binge drinking, psychological inflexibility, and distress tolerance. Psychological inflexibility was associated with greater cigarette dependence (*β* = .325, *p* = .013) and binge drinking (*β* = .303, *p* < .001). In linear but not zero-inflated negative binomial models, distress tolerance moderated the relationship between psychological inflexibility and binge drinking frequency (*β* = .160, *p* = .003), though not the relationship between psychological inflexibility and cigarette dependence. Findings highlight the role of psychological inflexibility in the use of cigarettes and alcohol among people who co-use cannabis for therapeutic purposes. This population may benefit from interventions aimed at reducing psychological inflexibility and improving distress tolerance.

The use of cannabis for therapeutic purposes is legal in 39 U.S. states, three U.S. territories, and the District of Columbia, while 24 states, three territories and the District of Columbia have enacted measures to regulate cannabis for non-medical, recreational use (National Conference of State Legislatures (NCSL), 2025). Research on the therapeutic benefits of cannabis is mixed, with the most support for chronic pain, chemotherapy-induced nausea and vomiting, and patient-reported spasticity symptoms associated with multiple sclerosis (National Academies of Sciences, Engineering, and Medicine, 2017). Potential undesirable effects associated with cannabis use include addiction, adverse brain changes, and impaired attention, memory, and learning (Volkow et al., 2014).

Adverse effects of cannabis use may be exacerbated in the context of the co-use of cannabis with other substances (Tucker et al., 2019; Yurasek et al., 2017). In fact, cannabis, cigarette, and alcohol consumption have been shown to increase the odds of same day co-use (i.e., using one of the other substances) and tri-use (i.e., using both of the other substances; Roche et al., 2019). Moreover, cannabis and cigarette co-use has been associated with cannabis use disorder onset, persistence, and relapse over time (Weinberger et al., 2021). In addition, cannabis and alcohol co-use has been associated with higher odds of alcohol use disorder (Waddell, 2022).

Individuals who use cannabis and cigarettes tend to report heavier cigarette and cannabis use in addition to worse overall physical and mental health compared to individuals who use only one or the other (Tucker et al., 2019). Additionally, co-use of cannabis and cigarettes may result in increased exposure to carbon monoxide, toxicants, and carcinogens, depending on the product and method of administration (e.g., smoking combustible products). Cannabis and alcohol co-use has also been associated with a number of adverse consequences, including impaired cognitive performance, behavioral and social consequences (e.g., legal issues, lower academic achievement, high-risk sexual behaviors, driving while intoxicated), risk of abnormal brain development in adolescence, and poor mental health (e.g., co-occurring substance use disorders, anxiety disorders, mood disorders; Yurasek et al., 2017). Cannabis use disorder, cigarette dependence, and alcohol use disorder are highly comorbid with one another and their co-use can interfere with efforts to reduce or quit each substance (McClure et al., 2020). For these reasons, it is important to understand potential modifiable risk factors that may contribute to the use of other substances, including cigarettes and alcohol, among people who use cannabis for therapeutic purposes.

Psychological inflexibility is characterized by an unwillingness to experience distressing feelings, emotions, or sensations, with attempts to avoid or control them (Hayes-Skelton & Eustis, 2020) and is one such modifiable factor. Specifically, this refers to one’s ability to “be present,” or to fully engage in whatever you are doing, to “open up,” or to allow oneself to experience one’s thoughts and feelings, and to “do what matters,” or to act in accordance with one’s values (Harris, 2022). Psychological inflexibility, a promising target in the treatment of substance use disorders (De Groot et al., 2014; Gloster et al., 2020), is the opposite of psychological flexibility and refers to one’s *inability* to fully experience the present moment without trying to change it or its accompanying thoughts and emotions (Hayes et al., 2006).

This inability has been associated with increased substance use (Farris et al., 2015a, 2015b; Luoma et al., 2020) as individuals commonly use substances to try to avoid or distract themselves from uncomfortable internal experiences (e.g., negative thoughts, feelings, sensations). In addition, it has been linked to a number of alcohol-related problems (e.g., problematic alcohol use, academic difficulties, interpersonal problems, high risk or illegal behavior, health consequences; Luoma et al., 2020) and cigarette smoking-related problems (e.g., cigarette dependence, difficulty quitting smoking, perceived barriers to quitting smoking, lower motivation to quit smoking, positive smoking expectancies, greater withdrawal, worse cravings; Buckner et al., 2015; Farris et al., 2015a, 2015b). Thus, psychological inflexibility represents an important risk factor and treatment target for the persistent use of substances, such as alcohol and cigarettes.

Despite research highlighting increased substance use severity and difficulties quitting among individuals who co-use cannabis with other substances (e.g., cigarettes, alcohol), there is minimal research on psychological inflexibility and substance co-use. Most existing research in this area has examined the role of psychological inflexibility and use of individual substances (e.g., cigarettes or alcohol) or substance use disorders in general as opposed to co-use of specific substances (e.g., cannabis and cigarettes; cannabis and alcohol). Moreover, to the authors’ knowledge, only one study has examined psychological inflexibility and cannabis use. Bordieri and colleagues found that psychological inflexibility moderated the relationship between cannabis use severity and posttraumatic stress symptom severity such that higher levels of posttraumatic stress were associated with greater risk of cannabis use disorder only among individuals with psychological inflexibility (Bordieri et al., 2014). Although this sample recruited individuals from a residential substance use treatment program, the authors did not specifically report on participants’ co-use of other substances. Nonetheless, this study highlights that one’s level of psychological inflexibility may differentially impact the relationships between other variables (e.g., posttraumatic stress) and cannabis use among individuals with substance use disorders.

Distress tolerance may also impact the relationship between psychological inflexibility and the co-use of cannabis with cigarettes or alcohol. Distress tolerance is considered conceptually distinct from psychological inflexibility. Distress tolerance refers to one’s perceived or actual ability to withstand negative internal states (Zvolensky, 2011), whereas psychological inflexibility refers to one’s unwillingness to contact the present moment and attempts to avoid, alter, or control negative emotional states. Thus, it is possible that an individual who views negative internal states as intolerable (i.e., low distress tolerance) may be highly motivated to avoid, reduce, or control them (i.e., demonstrate high psychological inflexibility) through various means, including by engaging in substance use. Distress tolerance is commonly associated with smoking cessation lapse (Veilleux, 2019), and is associated with problematic alcohol use both directly (Zegel et al., 2021) and indirectly through coping motives (Pilatti et al., 2022), physiological stress reactivity (Holzhauer et al., 2017), cognitive schemas (Simons et al., 2018), depressive symptoms (Brooks Holliday et al., 2016), and posttraumatic stress symptoms (Brooks Holliday et al., 2016).

Due to the high prevalence and negative consequences associated with the co-use of cannabis with cigarettes and alcohol, it is important to identify factors (e.g., psychological inflexibility, distress tolerance) that may relate to greater cigarette smoking and alcohol use among individuals who use cannabis for medical purposes. By identifying these factors, mental health professionals can design interventions to reduce the likelihood of substance co-use and be aware of potential therapeutic targets that may serve as a focus of psychotherapy to reduce distress and increase life satisfaction (Karekla et al., 2025; Weststrate et al., 2023). It is especially important to examine these factors in samples using cannabis for therapeutic purposes because policy changes have increased the use of cannabis among individuals already suffering from physical and psychiatric distress (Rhee & Rosenheck, 2023).

## Objectives

We hypothesized that higher psychological inflexibility would be associated with greater cigarette dependence and greater binge drinking frequency. The primary aims of the current study were therefore to:

- Examine the relationship between psychological inflexibility and cigarette dependence among individuals who co-use cannabis for therapeutic purposes and combustible cigarettes, and
- Examine the relationship between psychological inflexibility and binge drinking among individuals who co-use cannabis for therapeutic purposes and alcohol.
- An exploratory aim of this study was to examine whether distress tolerance moderated these relationships.

## Method

### Participants and Procedure

Participants were recruited from a licensed medical cannabis dispensary in New Jersey. Research assistants approached adults in the dispensary waiting room and invited them to participate in the study. Of the 1185 individuals who were approached, 705 agreed to participate (59.5% response rate). Data from 8 participants were unusable, resulting in a sample size of 697. Adults (ages 18–89) who could read and speak English were considered eligible for the study. Participants read the consent form and completed the survey on an iPad and were provided with a physical copy of the consent form for their records. Participants were entered in a raffle with a 1 in 25 chance of winning a $25 Amazon gift card as compensation for their time. At the time of the study (data were collected between February and September 2019), cannabis was legal in New Jersey for medical but not recreational purposes. The study was approved by the Institutional Review Board of Rutgers University (protocol number Pro2018001382).

### Measures

#### Demographics

Most demographic questions were selected from the National Health and Nutrition Examination Survey (NHANES) and included assessments of sex, race, ethnicity, marital status, education, employment status, and annual household income.

#### Substance Use Variables

Cannabis frequency, route of administration (i.e., smoked, aerosolized/“vaped”, ate, drank, dabbed, other), and medical marijuana qualifying condition were assessed. Current smoking status was defined as smoking at least 100 lifetime cigarettes and smoking within the last 30 days. Cigarette dependence was measured using the Heaviness of Smoking Index (HSI) (Heatherton et al., 1989), which includes two items (i.e., number of cigarettes per day, minutes until first cigarette of the day) with response options ranging from 0–3. Total HSI scores range from 0–6, with higher scores reflecting higher cigarette dependence. Binge drinking was assessed by asking participants, “On how many days in the past month did you consume five (5) or more drinks containing alcohol?”.

#### Psychological Inflexibility

Psychological inflexibility was measured using the total score on the Acceptance and Action Questionnaire (AAQ-II) (Bond et al., 2011). The AAQ-II is a 7-item scale with response options ranging from 1 (“never true”) to 7 (“always true”). Total scores are summed and range from 7–49, with higher scores on the AAQ-II reflect higher psychological inflexibility. The AAQ-II demonstrated good internal consistency in this sample, *α* =.937.

#### Distress Tolerance

The 15-item Distress Tolerance Scale (Simons & Gaher, 2005) was used to assess participants’ ability to tolerate negative psychological states. Responses range from 1 (“strongly agree”) to 5 (“strongly disagree”) and scores are averaged, with higher scores reflecting higher levels of distress tolerance. Possible scores, therefore, range from 1 to 4. The scale consists of 4 subscales (i.e., tolerance, appraisal, absorption, regulation), and a higher order global distress tolerance score is calculated by averaging the mean of each subscale. The Distress Tolerance Scale demonstrated good internal consistency in this sample, *α* =.925.

### Data Analytic Plan

Descriptive statistics (i.e., mean, standard deviation, frequency, percentage) were calculated for all study variables. Tests for homogeneity of variance (Levene’s statistic) indicated that assumptions were not violated (all p > 0.05) for t-tests or ANOVAs. In addition, skewness was between 0 and 0.5 indicating an approximately symmetrical distribution for variables included in Pearson correlations (i.e., age and Distress Tolerance Scale). Pearson correlations, *t*-tests, and analysis of variance were used to examine the relationship between psychological inflexibility and all study variables, and to assess for potential covariates.

We conducted multiple linear regression analysis adjusting for age, sex, employment, income, and marital status. Sex (male, female), employment (employed, unemployed), income ($10,000 or less, more than $10,000), and marital status (married, not married) were dummy coded prior to entry into the model. Psychological inflexibility, distress tolerance, and the interaction between psychological inflexibility and distress tolerance (to address the exploratory aim examining whether distress tolerance moderated these relationships) were included as independent variables. Psychological inflexibility and distress tolerance variables were centered prior to creating the interaction term and entering these variables in the model because of multicollinearity concerns. We also tested zero-inflated Poisson and negative binomial models as a sensitivity analysis for the analysis regarding binge drinking because 67.6% of participants denied any binge drinking in the past month. Independent variables and covariates were added to the model and separate analyses were conducted for each outcome variable (i.e., cigarette dependence, binge drinking frequency).

## Results

### Participant Characteristics

Participants were 697 adults, ages 18 to 86, recruited from a medical cannabis dispensary (60% male, 75% white). Additional participant characteristics, including mean scores on variables of interest, are displayed in Table 1. Participants reported using cannabis on 24 of the past 30 days on average (SD = 9.78), with 65% reporting daily use. Most (75%) participants reported smoking as their primary route of administration, followed by vaping (20%). Past-month combustible cigarette use was reported by 17% of participants, with 61.9% of these participants reporting daily cigarette use. Most participants denied binge drinking in the past month (67.6%), though binge drinking ranged from none to 25 days in the past month, with an average of once a month (M = 1.13, SD = 2.73).

### Psychological Inflexibility

As depicted in Table 2, demographic variables related to psychological inflexibility included age, *r* = −.237, *p* <.001, employment, *F*(7,635) = 6.290, *p* <.001, household income, *F*(7,635) = 5.441, *p* <.001, sex, *t*(642) = −2.368, *p* =.018, and marital status, *F*(4, 639) = 8.870, *p* <.001. Specifically, younger individuals (*r* = −.237, *p*, <.001), students (*M* = 29.57, *SD* = 10.56), individuals who were out of work for over one year (*M* = 28.52, *SD* = 10.97) or are unable to work (*M* = 27.17, *SD* = 12.16), those who made less than $10 K (*M* = 28.83, *SD* = 12.06), males (*M* = 22.61, *SD* = 10.78), and individuals who were divorced (*M* = 26.25, *SD* = 12.15) or never married (*M* = 25.56, *SD* = 10.63) had higher psychological inflexibility. In addition, greater psychological inflexibility was associated with lower distress tolerance, *r* = −.646, *p* <.001. Variables with statistically significant associations with psychological inflexibility were included as covariates in linear regression models examining psychological inflexibility and a) cigarette dependence and b) binge drinking.

### Psychological Inflexibility and Cigarette Dependence

Results of linear regression analysis indicated that psychological inflexibility (β =.325, SE =.015, *p* =.013) was related to greater cigarette dependence, while distress tolerance (β = −.109, SE =.164, *p* =.383) and the interaction between psychological inflexibility and distress tolerance (β =.007, SE =.009, *p* =.936) were not associated with cigarette dependence (see Table 3), in models adjusting for age, sex, employment, income, and marital status.

### Psychological Inflexibility and Binge Drinking

**Fig. 1:**
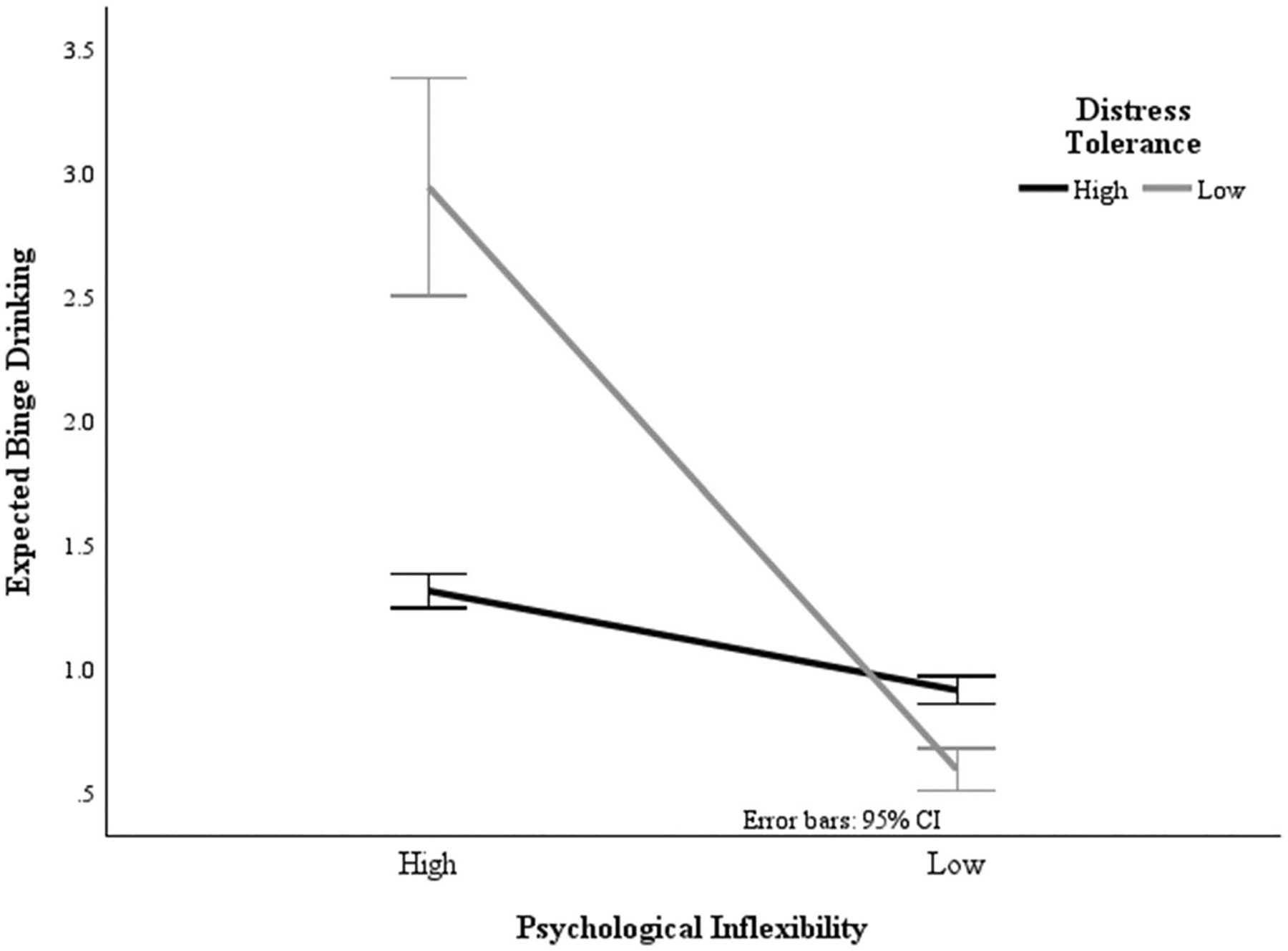
Interaction between psychological inflexibility and distress tolerance on binge drinking frequency. *Note*. *N* = 133; Binge drinking = 5 or more drinks in one sitting; Psychological Inflexibility measured by the Acceptance and Action Questionnaire (AAQ-II; Bond et al., 2011); Distress Tolerance measured by the 15-item Distress Tolerance Scale (DTS; Simons & Gaher, 2005)

Results of linear regression analysis indicated that the interaction between psychological inflexibility and distress tolerance (β =.160, SE =.014, *p* =.003) on binge drinking frequency was significant in models adjusting for age, sex, employment, income, and marital status. This finding suggests that distress tolerance moderated the relationship between psychological inflexibility and binge drinking frequency (see Table 4). While individuals with lower psychological inflexibility reported less frequent binge drinking overall, those with higher psychological inflexibility and lower distress tolerance reported the greatest binge drinking frequency (see Fig. 1).

**Fig. 2:**
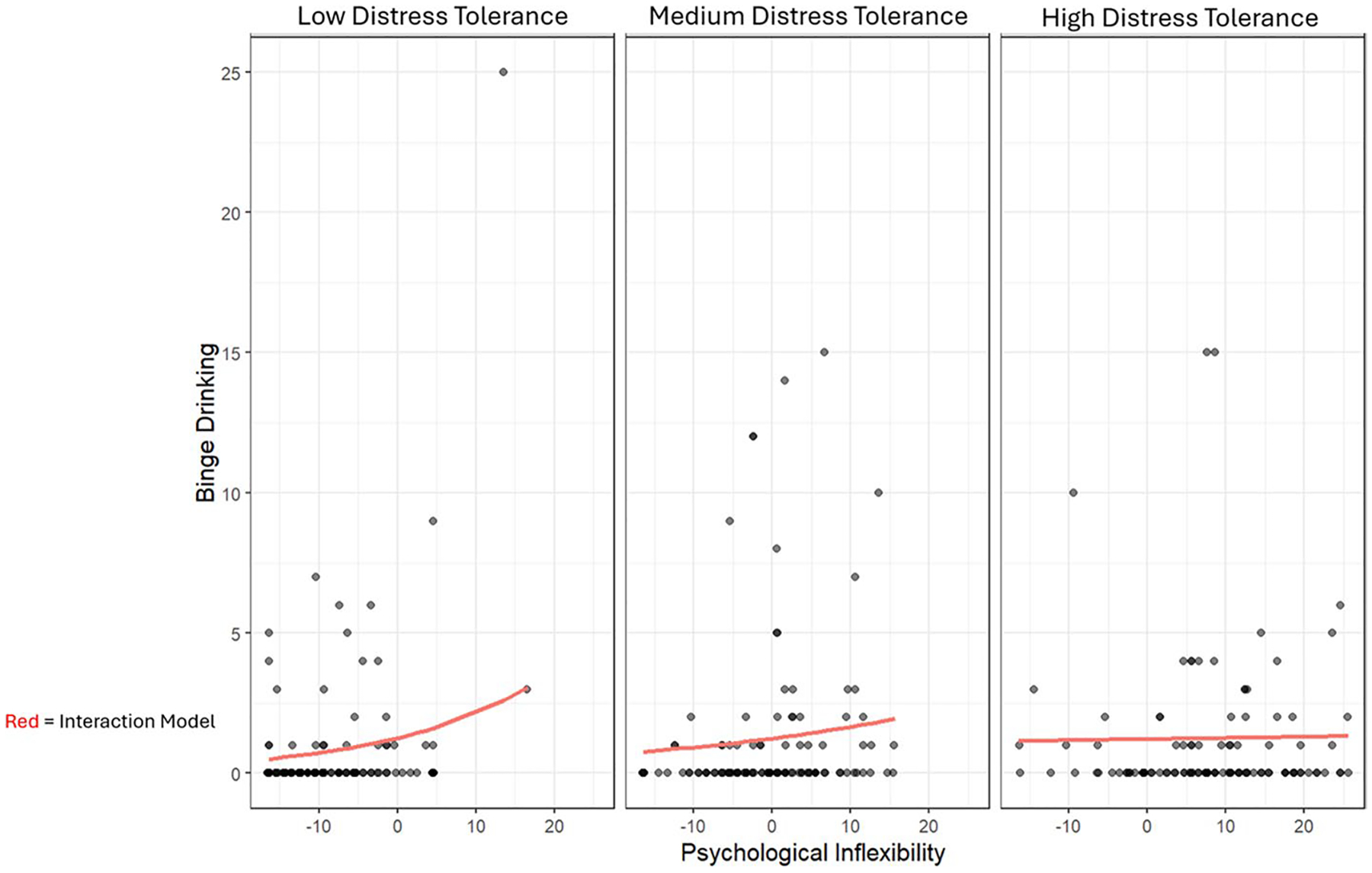
Zero-inflated negative binomial modeling of the interaction between psychological inflexibility and distress tolerance on binge drinking frequency. *Note*. *N* = 133; Binge drinking = 5 or more drinks in one sitting; Psychological Inflexibility measured by the Acceptance and Action Questionnaire (AAQ-II; Bond et al., 2011); Distress Tolerance measured by the 15-item Distress Tolerance Scale (DTS; Simons & Gaher, 2005)

To address concerns of zero-inflation (because 67.6% of participants denied any binge drinking in the past month), we conducted a sensitivity analysis by fitting the same proposed model as a zero-inflated Poisson model and as a zero-inflated negative binomial model. Model fit indices favored the zero-inflated negative binomial model (AIC = 817.79; BIC = 890.48; Bayes Factor > 100) over the Poisson model (AIC = 973.62; BIC = 1042.48; Bayes Factor = 0), therefore we used the negative binomial model as our zero-inflated model. When modeled as a zero-inflated negative binomial, the interaction between psychological inflexibility and distress tolerance did not achieve statistical significance (estimate = −0.024, *z*-value = −1.884, SE = 0.013; *p* = 0.059) (see visualizations in Fig. 2). Consistent with the linear model, however, individuals low in psychological inflexibility appeared to report less frequent binge drinking overall, while individuals high in psychological inflexibility *and* low in distress tolerance appeared to report the greatest binge drinking frequency.

## Discussion

The current study found that, among individuals who co-use cannabis for therapeutic purposes, alcohol, and cigarettes, psychological inflexibility was associated with greater cigarette dependence and greater binge drinking frequency. Moreover, distress tolerance moderated the relationship between psychological inflexibility and binge drinking frequency—with the caveat that zero-inflated modeling did not find quantitative support for moderation. Of note, consistent with hypotheses, visualizations of the zero-inflated negative binomial model do suggest an interaction may be present, such that, for individuals low in distress tolerance, greater psychological inflexibility was associated with increased binge drinking, while for individuals high in distress tolerance no such relationship appeared to be present (see Fig. 2. Specifically, the relationship between psychological inflexibility and binge drinking frequency was strongest among individuals who reported lower distress tolerance.

Psychological inflexibility was associated with cigarette dependence and binge drinking among individuals who co-use cannabis for therapeutic purposes; thus, decreasing one’s level of psychological inflexibility may be particularly beneficial in targeting and reducing problematic substance use in this population. Doing so has the potential to increase individuals’ mindfulness and acceptance of uncomfortable internal states (e.g., craving), without avoiding or dampening them with substances, as they engage in activities that provide meaning and purpose (Shorey et al., 2017). In line with this, decreased psychological inflexibility has been associated with positive treatment outcomes (e.g., lower cravings, reduced addiction severity, discontinued substance use) for a range of substances, including cigarettes (Buckner et al., 2015) and alcohol (Byrne et al., 2019). Future research should examine whether interventions that target psychological inflexibility produce positive treatment outcomes among individuals who co-use cannabis with cigarettes and/or alcohol.

Of note, distress tolerance moderated the relationship between psychological inflexibility and binge drinking frequency but not cigarette dependence. There are no previous studies examining this moderating relationship; however, multiple possibilities may explain this discrepancy. Although binge drinking may contribute to the severity of alcohol dependence, measuring binge drinking frequency is likely to be meaningfully different than measuring actual dependence, as we did with cigarette use. In addition, our general measure of psychological flexibility (Bond et al., 2011) may not have been ideal for these purposes. Because we collected data on a variety of substances, we chose a measure of general, rather than content-specific, psychological flexibility. Our findings may have differed if we had examined substance-specific psychological flexibility, or experiential avoidance for different substances by using a measure such as the Avoidance and Inflexibility Scale (Farris et al., 2015a, 2015b) for analyses related to cigarette smoking and the alcohol specific version of the Acceptance and Action Questionnaire – Substance Abuse (AAQ-SA; Luoma et al., 2011) for analyses related to alcohol use. Additionally, the lack of a statistically significant relationship between distress tolerance and cigarette dependence is inconsistent with some previous work (Bello et al., 2023; Garey et al., 2024; Schlam et al., 2019), which found an inverse relationship. Another group (Niezabitowska et al., 2022), which, like us, found no direct relationship between nicotine dependence and distress tolerance, did find an indirect relationship through smoking motives. It is possible that other, unmeasured variables may also serve as mediators in our data.

The role of distress tolerance has not yet been examined in the relationship between psychological inflexibility and cigarette use, alcohol use, or their co-use with cannabis. However, one study has examined whether distress tolerance and psychological inflexibility are independently associated with substance use cravings in general (Shorey et al., 2017). In a residential substance use treatment sample, lower psychological inflexibility and higher distress tolerance were associated with lower substance use cravings when examined individually; however, only psychological inflexibility remained associated with cravings when examined simultaneously with distress tolerance (Shorey et al., 2017). Shorey and colleagues (2017) did not examine the interaction between psychological inflexibility and distress tolerance; thus, it remains unclear whether distress tolerance moderated the relationship between psychological inflexibility and substance use cravings in their sample.

The current study adds to the literature by examining distress tolerance as a potential moderator in the relationship between psychological inflexibility and problematic substance use. It highlights that the role of distress tolerance may differ depending on the type of substance (e.g., alcohol vs. cigarette use) or measure (e.g., behavioral outcome vs. dependence). Given how little binge drinking was endorsed in our sample, future research may seek to replicate this finding in a sample with a greater percentage of participants endorsing binge drinking.

### Limitations

These findings should be interpreted in the context of several limitations. Participants were recruited from a single dispensary (though in an ethnically and racially diverse community) and may not be representative of medical dispensary patrons from other geographic areas. Moreover, it was not possible to invite every consecutive patron of the dispensary, and there may be meaningful differences between those who agreed to complete the survey and those who declined, thus limiting the generalizability of our findings. Additionally, the current study was a secondary analysis of cross-sectional data, which restricts the ability to infer causal associations (e.g., whether psychological inflexibility leads to greater cigarette dependence or binge drinking frequency over time). Moreover, there are questions about the validity of the AAQ-II as a measure of psychological inflexibility versus negative affect (McLoughlin & Roche, 2022). Finally, the measures used in this study relied on self-report and could be subject to social desirability, recall bias, and under-reporting.

### Conclusion

The current study reports on people who use cannabis for therapeutic purposes and there may be differences in these relationships among people using cannabis for medical vs. non-medical purposes. Findings from the current study highlight the role of psychological inflexibility in the co-use of cannabis with cigarettes and alcohol. Individuals who use cannabis for therapeutic purposes may at times engage in heavy cigarette smoking and binge drinking to escape or avoid uncomfortable internal states related to the physical and mental health conditions for which they are prescribed medical cannabis (e.g., pain, anxiety). This may be particularly true among those with lower levels of distress tolerance.

This has important clinical implications, as it suggests the need for screening, and if appropriate, empirically supported intervention. Specifically, screening and interventions aimed at decreasing psychological inflexibility and improving distress tolerance may support individuals who co-use cannabis for therapeutic purposes and wish to reduce alcohol or cigarette use. The provision of interventions which increase psychological flexibility, such as Acceptance and Commitment Therapy (ACT) (Hayes et al., 2006) for the treatment of alcohol (Byrne et al., 2024) and nicotine dependence (Santiago-Torres et al., 2023) should be considered for individuals using cannabis for therapeutic purposes wishing to stop co-using cigarettes and/or alcohol. In contrast to this secondary analysis of cross-sectional data, future research should prospectively examine these relationships. Additionally, the field would benefit from randomized clinical trials prospectively assigning participants using cannabis for therapeutic purposes wishing to change their alcohol and/or cigarette use to psychosocial interventions focused on psychological flexibility (i.e., ACT) or, as a comparison, to interventions targeting other constructs.

## Figures and Tables

**Table 1:** Participant characteristics and study variables

Variable	M (SD) or N (%)
Age	41.97 (14.33)
Gender	
Male	387 (60.0%)
Female	252 (39.1%)
Gender nonconforming	5 (0.8%)
Ethnicity	
Hispanic, Latino/a, or Spanish	93 (14.9%)
Not Hispanic, Latino/a, or Spanish	551 (85.1%)
Race	
White	525 (75.3%)
Black	59 (8.5%)
American Indian/Alaska Native	17 (2.4%)
Asian	16 (2.3%)
Native Hawaiian/Pacific Islander	5 (0.7%)
None of the above	9 (1.3%)
Annual Household Income	
Less than $10 K	58 (9.0%)
Less than $15 K ($10 K—< $15 K)	25 (3.9%)
Less than $20 K ($15 K—< $20 K)	29 (4.5%)
Less than $25 K ($20 K—< $25 K)	31 (4.8%)
Less than $35 K ($25 K—< $35 K)	58 (9.0%)
Less than $50 K ($35 K—< $50 K)	68 (10.6%)
Less than $75 K ($50 K—< $75 K)	95 (14.8%)
$75 K or more	279 (43.4%)
Employment	
Employed for wages	316 (49.1%)
Self-employed	90 (14.0%)
Out of work for 1 + years	21 (3.3%)
Out of work for < 1 year	15 (2.3%)
Homemaker	18 (2.8%)
Student	28 (4.4%)
Retired	54 (8.4%)
Unable to work	101 (15.7%)
Highest education completed	
0–11th grade	23 (3.6%)
High school	169 (26.3%)
1 st year college/university	80 (12.4%)
2nd year college/university	100 (15.6%)
3rd year college/university	38 (5.9%)
5th + year college/university	113 (17.6%)
Marital status	
Never married	253 (39.3%)
Married	271 (42.1%)
Separated	17 (2.6%)
Divorced	70 (10.9%)
Other/unknown	33 (5.1%)
Qualifying Condition	
Amyotrophic Lateral Sclerosis (ALS)	1 (0.1%)
Anxiety	425 (61.0%)
Opoiod Use Disorder	30 (4.3%)
Post-Traumatic Stress Disorder	150 (21.5%)
Chronic pain musculoskeletal origin	258 (37.0%)
Chronic pain visceral origin	70 (10.0%)
Migraine	112 (16.1%)
Multiple Sclerosis	22 (3.2%)
Terminal cancer	10 (1.4%)
Non-terminal cancer	35 (5.0%)
Muscular Dystrophy	10 (1.4%)
Inflammatory Bowel Disease (IBD)	77 (11.0%)
Non-cancer terminal illness	7 (1.0%%)
Tourette’s	5 (0.7%)
Seizure	23 (3.3%)
Intractable skeletal muscular spasticity	32 (4.6%)
Glaucoma	15 (2.2%)
Human Immunodeficiency Virus	6 (0.9%)
Acquired Immunodeficiency Syndrome	4 (0.6%)
Binge drinking days per month	1.59 (3.7)
AAQ-II total score	23.39 (11.0)
DTS Global score	3.21 (1.0)

Qualifying condition refers to medical or psychiatric reason for cannabis use

*AAQ-II* Acceptance and Action Questionnaire (Bond et al., 2011), *DTS* Distress Tolerance Scale (Simons et al., 2005); One male and one female participant identified as transgender

**Table 2:** Relationship between total score on the Acceptance and Action Questionnaire-II (AAQ-II) and participant characteristics

	Mean	SD	η^2^ (95% CI)	Cohen’s d (95% CI)	*r*
Employment***			0.07 (0.03,0.10)		
Employed for wages ^a,b^	22.71	10.26			
Self-employed ^c^	20.02	10.32			
Out of work for ≥ 1 year ^c^	28.52	10.97			
Out of work for < 1 year	27.13	12.10			
Homemaker	24.06	9.42			
Student ^a,b,c,d^	29.57	10.56			
Retired ^d^	20.24	10.18			
Unable to work ^a,b,c^	27.17	12.16			
Income***			0.06 (0.02, 0.09)		
Less than $10,000 ^a^	28.83	12.06			
$10,000 to < $15,000	25.48	11.16			
$15,000 to < $20,000	26.00	10.56			
$20,000 to < $25,000 ^b^	28.00	11.87			
$25,000 to < $35,000	25.83	12.28			
$35,000 to < $50,000 ^a^	21.87	10.49			
$50,000 to < $75,000	23.27	10.90			
$75,000 or more^a,b^	21.34	9.81			
Biological Sex**				−0.19 (−0.35, −0.03)	
Male	22.61	10.78			
Female	24.68	11.08			
Marital Status***			0.05 (0.02, 0.09)		
Never married ^a^	25.57	10.63			
Married ^a,b^	20.53	10.15			
Separated	24.06	11.73			
Divorced ^b^	26.26	12.15			
Other/Unknown	24.70	11.51			
Age					−0.237***
DTS					−0.646***

Superscripts within levels of a variable denote statistically significant differences (*p* < 0.05). *DTS* Distress Tolerance Scale

** *p* < 0.01,

*** *p* < 0.001

**Table 3:** Results of linear regression model used to predict cigarette dependence (HSI)

	Coeff	CI	β	SE	*p*
Constant	2.643	(1.487, 3.800)		.584	<.001
Age	−0.008	(−0.030, 0.015)	−.069	.011	.494
Sex (M, F)	0.315	(−0.170, 0.801)	.115	.245	.200
Employed (Y, N)	−0.309	(−0.808, 0.191)	−.113	.252	.223
Income (≤ $10 K, > $10 K)	−0.461	(−1.169, 0.247)	−.117	.358	.200
Married (Y, N)	0.462	(−0.088, 1.012)	.167	.278	.099
AAQ-II	0.038	(0.008, 0.068)	.325	.015	.013*
DTS	−0.144	(−0.469, 0.181)	−.109	.164	.383
AAQ-II × DTS	0.001	(−0.018, 0.019)	.007	.009	.936

*N* = 133, *HSI* Heaviness of Smoking Index (Heatherton et al., 1989), *AAQ-II* Acceptance and Action Questionnaire (Bond et al., 2011), *DTS* Distress Tolerance Scale (Simons et al., 2005); *R*^2^ =.116

* *p* <.05

**Table 4:** Results of linear regression model used to predict binge drinking frequency

	Coeff	CI	β	SE	*p*
Constant	0.090	(−1.512, 1.693)		.815	.912
Age	0.004	(−0.023, 0.031)	.021	.014	.763
Sex (M, F)	0.725	(0.139, 1.311)	.131	.298	.015
Employed (Y, N)	0.195	(−0.409, 0.798)	.035	.307	.526
Income (≤ $10 K, > $10 K)	0.286	(−1.147, 1.719)	.022	.728	.695
Married (Y, N)	0.671	(−0.039, 1.382)	.121	.361	.064
Psychological Inflexibility (AAQ-II)	0.081	(0.043, 0.119)	.303	.019	<.001***
Distress Tolerance (DTS)	−0.343	(−0.749, 0.063)	−.120	.207	.098
AAQ-II × DTS	−0.041	(−0.069, −0.014)	.160	.014	.003**

*N* = 133, *Binge drinking* = 5 or more drinks in one sitting; *AAQ-II* Acceptance and Action Questionnaire (Bond et al., 2011), *DTS* Distress Tolerance Scale (Simons et al., 2005); *R*^2^ =.083

** *p* <.01;

*** *p* <.001
